# Retrovirus Integrase-DNA Structure Elucidates Concerted Integration Mechanisms

**DOI:** 10.3390/v2051185

**Published:** 2010-05-10

**Authors:** Duane Grandgenett, Sergey Korolev

**Affiliations:** 1 Saint Louis University School of Medicine, Institute for Molecular Virology, 1100 S. Grand Blvd., St. Louis, MO 63104, USA; 2 Department of Biochemistry and Molecular Biology, 1100 S. Grand Blvd., St. Louis, MO 63104, USA; E-Mail: korolevs@slu.edu

## Abstract

Commentary on Hare, S.; Gupta, S.S.; Valkov, E.; Engelman, A.; Cherepanov, P. Retroviral intasome assembly and inhibition of DNA strand transfer. *Nature* **2010**, *464*, 232–236.

In the 11 March 2010 issue of Nature, Peter Cherepanov and colleagues from the Imperial College, London, determined the crystal structure of the prototype foamy virus (PFV) integrase (IN) in complex with its viral DNA [1]. IN has multiple roles in the retrovirus life cycle. In virus-infected cells, IN is located in the cytoplasmic preintegration complex (PIC) where it cleaves a dinucleotide from the 3’ OH DNA blunt-ends. Upon nuclear transport, IN promotes the concerted integration of the recessed linear DNA ends into opposite strands of the selected host site. The insertion of the viral DNA into the host genome results in a small duplication of cellular DNA sequences.

The Nature paper by Cherepanov and colleagues has provided the first atomic resolution view of a DNA-bound structure of four PFV IN monomers (Figure 1). The complex is termed intasome [2]. The crystal structure captures a functional intasome possessing two 3’ OH recessed DNA ends which is capable of concerted integration. In this structure, the non-transferred viral DNA strand is 19 nucleotides in length and the transferred strand is 17 nucleotides. The two transferred 3’ recessed ends are juxtaposed within the active catalytic site of IN (Figure 1A). The arrangement of the D,D(35)E motif in IN [3] with divalent metal ions as cofactors in the active site clearly supports S_N_2-type nucleophilic substitution during polynucleotidyl transferase functions, highly conserved throughout the retrovirus IN superfamily that includes transposases [4]. The active sites are located close to the shallow concave surface of the assembly suitable for binding a target DNA molecule (Figure 1B). The distance between the reactive 3’ ends corresponds to the expected distance between the integration sites into target DNA (4 base pairs) [5]. Therefore, the PFV intasome fits the conformation in the functional PIC.

The structure-based amino acid sequence alignment of PFV IN with HIV-1 IN demonstrates high conservation of key structural elements and provides a strong framework to utilize their new atomic resolution data for further development of HIV-1 IN inhibitors. The study is of great importance to virologists and is medically important because HIV-1 IN is an attractive and proven target for drug intervention. The paper provides interesting insights into biological functions of IN necessary for integration and effectiveness of clinically relevant inhibitors.

A remarkable feature of IN is an extremely elaborate network of protein-protein and DNA-protein interactions (Figure 1). IN is composed of three structural domains, the N-terminal domain (NTD), catalytic core domain (CCD), and C-terminal domain (CTD). Two monomers form all contacts with the viral DNA, and each of these monomers interacts with both DNA molecules. Two monomers form an elongated structure with dimensions of approximately 110 x 60 Å. Domains of each subunit are stretched out along the entire length of the extended assembly with the NTD and CCD positioned at remote opposite ends with the CTD sitting in-between. This arrangement results in extensive interface interactions between the NTD and CCD domains of opposite monomers. Finally, a small domain visualized in the structure, referred to as the NTD extension domain (47 residues and not shown in Figure 1), is located prior to the NTD and by sequence analysis found in other spumaviral IN species [1].

The whole elongated assembly of the two monomers is organized around two DNA molecules and is unlikely to exist in a DNA-free form. The 5’ overhang of the non-transferred DNA strand is threaded between the CTD and CCD of the monomer that also binds the 3’ end of the transferred strand of the same DNA molecule (Figure 1). As a result, the non-transferred strands are directed away from the potential binding site for the host DNA making this conformation ideal for the integration reaction. About half of a turn of double helix is sandwiched between two monomers involving the CCD of one monomer and the CTD and NTD of another. The remaining one and half turns of the double stranded DNA interacts mainly with the NTD of the opposite monomer. The stretched-out arrangement of the IN domains around two DNA substrates may explain the fact that none of the domain-domain interactions reported in previously solved protein structures resemble interactions in the DNA-bound dimer. The complexities of this interface network make it impossible to predict domains conformation based on mutagenesis and biophysical approaches. The acquired atomic resolution of the functional intasome is absolutely essential to understand the mechanisms involved in the concerted integration reaction catalyzed by IN.

At the same time, the multifunctional nature of IN which performs different reactions in the multiple steps of viral DNA processing and integration suggests that IN can adopt different conformations and oligomeric states. The long flexible interdomain linkers suggest that such alternative forms can significantly deviate from the one shown in Figure 1. The previously defined DNA-free form structures of IN are valuable in defining other structural and function roles that IN has in the retrovirus replication cycle, like interacting with cellular cofactors for selection of integration sites [6]. Additional crystal structures of IN captured at different steps of the integration reaction will be of a great importance.

Interestingly, the present complex does retain one of previously observed interdomain interface, although peripheral to all DNA binding sites. Each CCD of the DNA-bound dimer, or inner dimer, also interacts with the CCD of an additional monomer resulting in an overall tetrameric assembly state of IN (outside monomers not shown in Figure 1). The importance of this dimer interface for HIV-1 IN activity is documented (references in *4*). At the same time, the outer monomers are far from the DNA binding and active sites making it difficult to predict the functional role of these subunits in intasome assembly. Although additional CCDs are part of full-length molecules, the NTDs and CTDs of these molecules are disordered and are not visible in the presented structure. These features leave unanswered the question about the documented necessity of a tetrameric form of HIV-1 IN (*7–8*) and the role of these outer monomers in the concerted integration reaction. The authors speculate these PFV IN monomers may be involved in binding with host DNA. Likewise, one can speculate they may interact with viral DNA as well. With larger size viral DNA substrates (>1 kb) in complexes capable of concerted integration, the length of viral DNA protected by HIV-1 IN varies from ∼16 [8] to 32 [9] base pairs, depending on the conditions of assembly, the step of DNA processing, and the presence of strand transfer inhibitors. The outer monomers would be good candidates to form polymer-like assembly of IN on viral DNA to explain the extended protection of DNA by IN. Other IN subunits may also extend protection internally. Molecular studies of the HIV-1 PIC (*10–11*) suggest the association of IN with the viral DNA extends several hundred nucleotides from the termini.

The most medically relevant information produced by these authors was the study of Raltegravir (MK-0518) which was approved by the FDA for treatment of HIV/AIDS and Elvitegravir (GS-9137) used in clinical trials. They previously demonstrated that these inhibitors were capable of blocking the concerted integration reaction catalyzed by PFV IN [5]. Naturally, they addressed the question whether these inhibitors were capable of being bound by IN within the intasome and what new structural information would flow from such an important experiment. The soaking of preformed crystals of the intasome with these drugs demonstrated interactions of each inhibitor with specific residues of the active site, the invariant CA dinucleotide located at the 3’ OH recessed DNA end, and the divalent metal cofactors. Significantly, these associations resulted in the movement of the 3’ OH DNA ends from the active site by 6 Å, compared to the position of the 3’ end in the non-inhibitor bound PFV IN. They concluded both inhibitors have similar modes of binding and action that involved an induced fit mechanism.

In summary, the structure of the PFV intasome and its complexes with strand transfer inhibitors is the culmination of their extended efforts to obtain atomic resolution data of a complex capable of concerted integration. This structure represents a major advance in understanding the DNA-protein contacts and interdomain interfaces of IN within the intasome. Their contributions have and will promote advances for producing new inhibitors directed against HIV-1 IN and IN mutants that arise in patients treated with Raltegravir. In the future, it will be very important to obtain HIV-1 IN-DNA structures produced in the present of IN inhibitors.

## Figures and Tables

**Figure 1.**
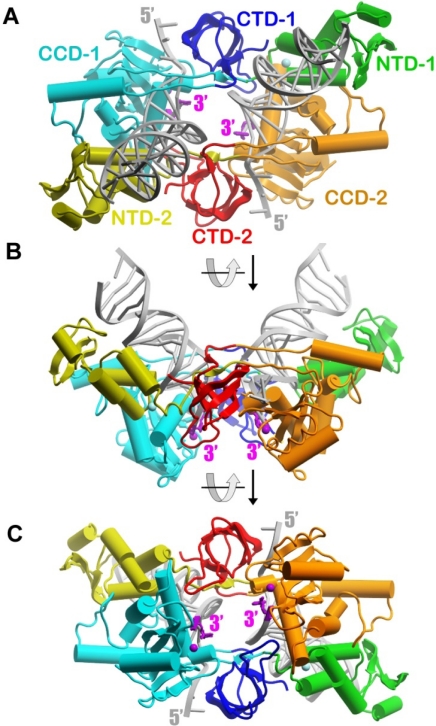
Schematic representation of the DNA-bound active subunits in the PFV intasome with the CCDs of outer monomers omitted for clarity (PDB ID: 3L2R [1]). **(A)** The NTD, CCD, and CTD of one monomer are shown in green, cyan, and blue, respectively. Analogous domains of the second monomer are colored as yellow, orange, and red. Helixes are shown as cylinders and strands as ribbons. The two DNA molecules are shown in grey. The reactive 3’ nucleotide is highlighted in magenta. The purple sphere is Mg^2+^. Orientations of the assembly in **(B)** represent a 90 degree rotation around the horizontal axis of the orientation in A). Correspondingly, the orientation in **(C)** is a 90 degree rotation from B). The picture was created with a ICM browser program.
